# Overview of Probe-based Storage Technologies

**DOI:** 10.1186/s11671-016-1556-9

**Published:** 2016-07-25

**Authors:** Lei Wang, Ci Hui Yang, Jing Wen, Si Di Gong, Yuan Xiu Peng

**Affiliations:** School of Information Engineering, Nanchang HangKong University, Nanchang, 330063 Peoples’ Republic China

**Keywords:** Probe, Density, Storage medium, Memory, Data

## Abstract

The current world is in the age of big data where the total amount of global digital data is growing up at an incredible rate. This indeed necessitates a drastic enhancement on the capacity of conventional data storage devices that are, however, suffering from their respective physical drawbacks. Under this circumstance, it is essential to aggressively explore and develop alternative promising mass storage devices, leading to the presence of probe-based storage devices. In this paper, the physical principles and the current status of several different probe storage devices, including thermo-mechanical probe memory, magnetic probe memory, ferroelectric probe memory, and phase-change probe memory, are reviewed in details, as well as their respective merits and weakness. This paper provides an overview of the emerging probe memories potentially for next generation storage device so as to motivate the exploration of more innovative technologies to push forward the development of the probe storage devices.

## Review

### Introduction

Today, the prosperity of social networks such as Youtube, Twitter, and Facebook, in conjunction with the digitalization of the daily service of worldwide citizens, has triggered a radical increase on the total amount of global digital data. According to IDC’s report, the amount of digital data has already surpassed 4.4 ZettaBytes (ZB) in 2013, and is predicted to reach 44 ZettaBytes in 2020 [1]. Under this situation, the capacities of various data storage devices where the digital data can be recorded and replayed are conversely limited in TeraBytes (TB) regime, lagging far behind the phenomenal growth of the digital data. The conventional mass storage devices can be categorized into hard disk, optical disc, and flash memory. Today, the hard disk drive (HDD) is undoubtedly the most famous mainstream storage device mainly due to its high capacity, fast data rate, and low cost. Nevertheless, in order to satisfy the current storage demand, the magnetic grain inside the HDD needs to be further shrunk so as to enhance the resulting density. However, the reduction on the volume of the magnetic grain would deteriorate the thermal stability of the magnetic written bits whose magnetization may be reversed without magnetic field due to the thermal fluctuation. This is well-known as superparamagnetic limit [2, 3]. The evolution of the optical disc has experienced three generations ranging from compact disc (CD) to Blu-ray disc. The major hurdle to prevent optical disc from booming its capacity results from the fact that the diameter of the laser spot focused on the optical disc is approximately proportional to the wavelength of the laser beam and inversely proportional to the numerical aperture (NA) of the objective lens [4, 5]. In this case, the storage capacity of the optical disc can seemingly be enhanced by either reducing the beam wavelength or increasing the NA. However, as there is no breakthrough progress recently made in the technologies of solid state laser and objective lens, the shortest wavelength and the largest NA to date are still limited to 405 and 0.85 nm for optical recording [6], which is usually referred to optical diffraction. Flash memory, represented by universal serial bus (USB) drive and iPhone series products, has recently received numerous attentions because of its high capacity, short data access time, non-volatility, portability, and low expense, which is expected to replace HDD in the near future. The capacity improvement of the flash memory strongly depends on the downscale of the flash cell size. In this case, further scaling down the cell size would result in a decrease on the thickness of the tunnel oxide layer usually sandwiched between the floating gate and wafer. A tunnel oxide layer with a very thin thickness is unable to prevent electrons from escaping from the floating gate, thus causing the loss of data, which is known as scaling limit [7, 8]. Consequently, as HDD, optical disc, and flash memory are suffering from superparamagnetic limit, diffraction limit, and scaling limit, it is timely to explore more innovative mass storage devices with higher storage capacity, faster data rate, lower energy consumption, and longer data retention time than conventional counterparts, giving rise to the advent of probe-based storage devices.

**Fig. 1:**
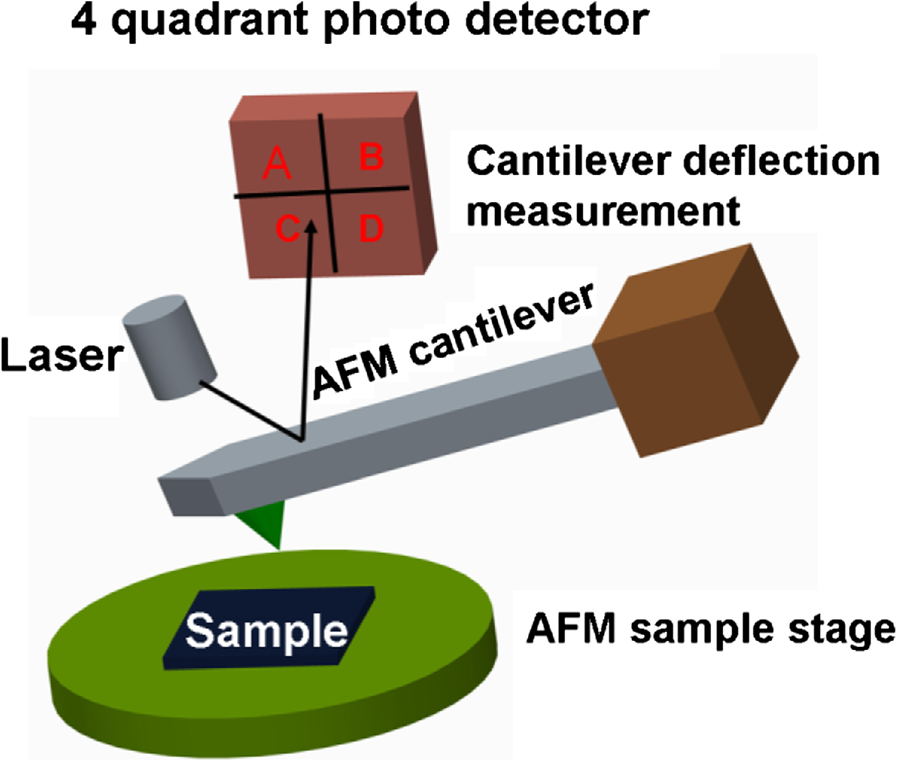
Schematic of AFM probe

**Fig. 2:**
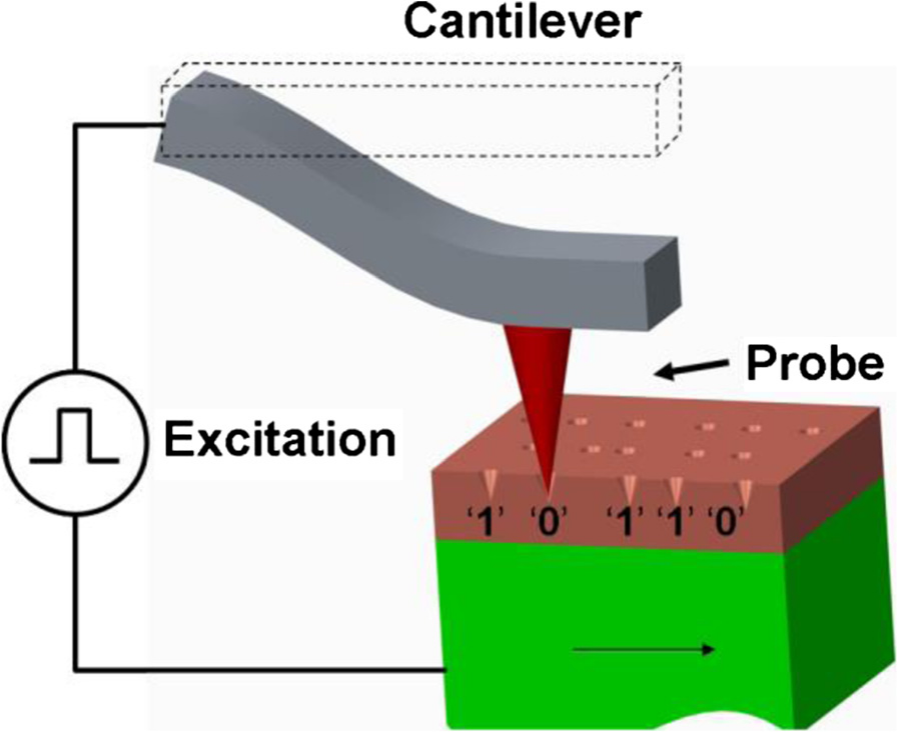
Principle of probe-based storage memory

A probe-based storage device can be defined as a storage memory that utilizes nanoscale probe to record and read bits in the storage medium. Prior to the probe-based storage device, nanoscale probe has been widely used for scanning probe microscope (SPM) applications to map out the tomography image of the sample [9]. To achieve this, nanoscale probe usually makes use of a cantilever with a very sharp tip integrated on its end to perform the raster scanning in which tip is brought closely to or in contact with sample. During the scanning, the tip can be deflected up or down due to the variation of the atomic force between tip apex and sample surface, and tomography image of sample surface can therefore be constructed by measuring the position of the tip, as schematically shown in Fig. 1. For probe-based storage device, an external excitation that is initially applied to the nanoscale probe can be employed to change the physical properties of the storage medium when in contact with the probe tip during the scanning, and thereby the region subjected to the external excitation exhibits different physical properties from the region without suffering from the excitation. As a result, these two regions with different characteristics can be regarded as the binary bits “1” and “0”, respectively, to realize the storage function. The principle of the probe-based storage device is illustrated in Fig. 2. According to the descriptions above, the size of the recorded bit is primarily determined by the size of the tip apex used for recording process, implying a fact that the implementation of tip apex on the order of nanometers would allow for an areal density over multi Terabit per square inch (Tbit/in^2^). In addition, the small size of recorded bit can lead to an aggressive reduction on the required energy consumption. Thanks to these merits, worldwide researchers have been dedicated to exploring probe-based storage technologies using various storage materials and system architectures, and this gives rise to an emergence of a variety of innovative probe memories such as thermo-mechanical probe memory, magnetic probe memory, ferroelectric probe memory, and phase-change probe memory. In this case, it is essential to have a thorough review on the physical principles and current status of various probe-based storage devices to help scientists to understand the inherent physics of these devices. However, such review articles are rarely found according to Web of Science by the time of writing and the latest review article about probe storage technologies has been dated back to 2011 [10]. As a result, a comprehensive review concerning probe-based storage devices is urgently required so as to reveal the physical mechanisms of these devices, and thus to ignite the research enthusiasm to further mitigate their respective storage performances.

### Thermo-Mechanical Probe Memory

**Fig. 3:**
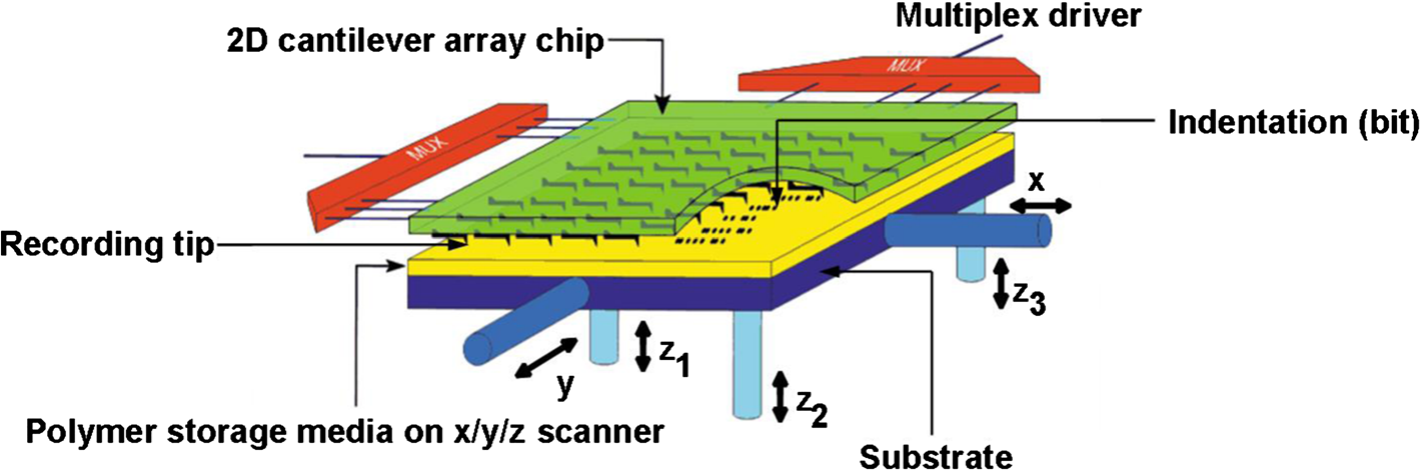
Millipede system. Reprinted with permission from [11]

**Fig. 4:**
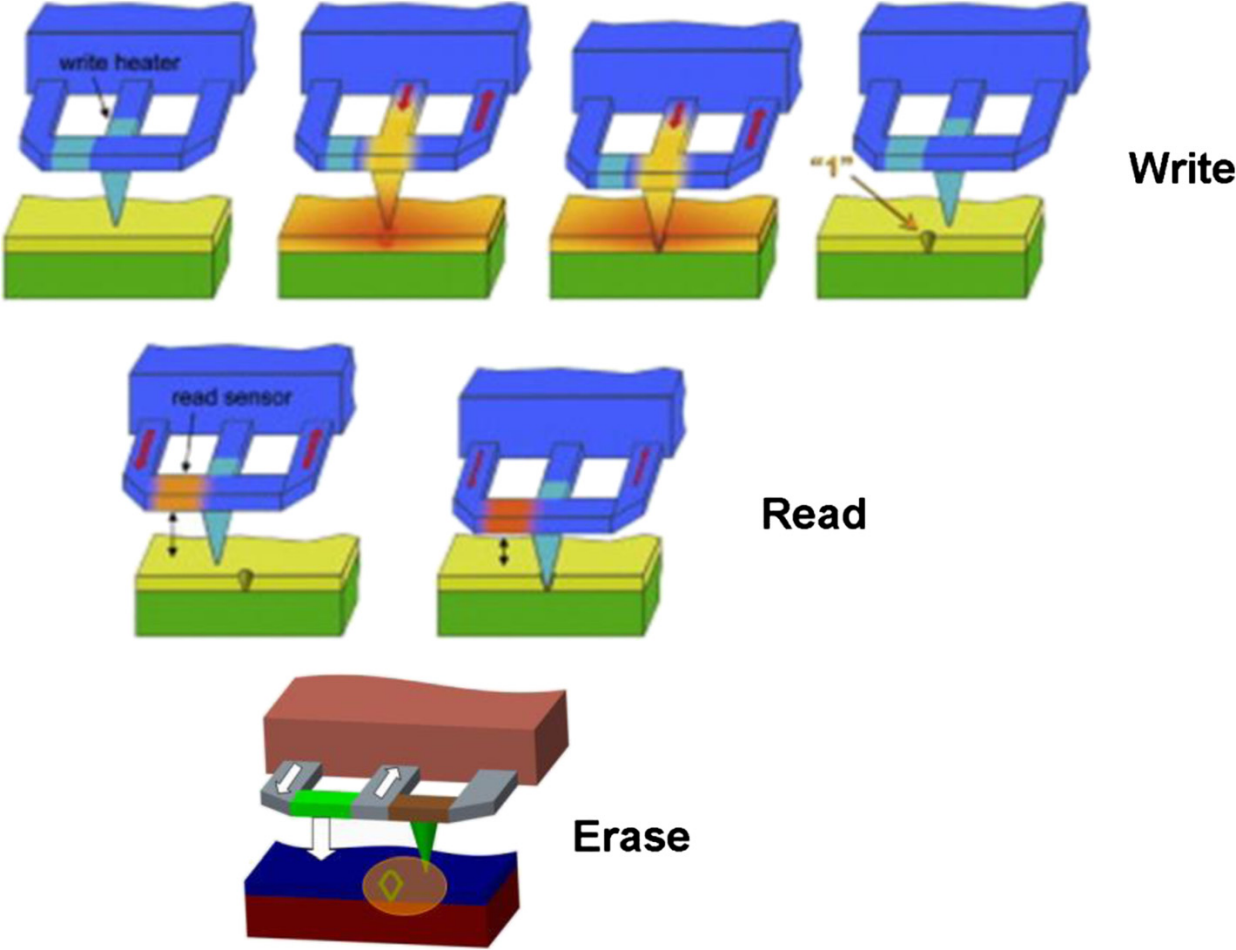
Millipede memory when operated in write mode, read mode, and erase mode. Reprinted with permission from [13]

The principle of thermo-mechanical probe memory is to pull a heated tip into the polymer medium to generate topographic change that can be denoted as binary digit “1” on medium surface [11]. The driving force behind the development of thermo-mechanical probe memory can be ascribed to the presence of IBM’s Millipede system that consists of an array of SPM tips with a moving storage medium underneath [11], as shown in Fig. 3. A localized heater, usually made of low-doped resistive silicon is integrated at the tip base, acted as heating element. During the writing process, an electrostatic force is applied to the cantilever to push the tip that is heated around polymer’s glass transition temperature (400 °C) into the polymer medium to soften it. The heated polymer medium would melt and allow tip to penetrate into it, thus generating an indentation on medium surface. This indentation and surrounding continuous flat surface can be used to represent “1” and “0”, respectively. The erasing process can be achieved by removing the applied force to pull the tip up back from the indentation. This would result in a surface tension to flatten the previously written indentation [12]. During the readout process, a read resistor whose resistance strongly depends on the temperature is integrated in one of the side-arms of the three-legged cantilever design, which is heated to around 300 °C. When tip falls into an indentation, the distance between tip and substrate is reduced, thereby accelerating heat leaking through the substrate. In this case, the temperature of the read resistor is changed due to the cooling process, and this would change the resistance of the read sensor, which can be detected as readout signals. The writing, readout, and erasing modes of thermo-mechanical probe memory are schematically shown in Fig. 4.

**Fig. 5:**
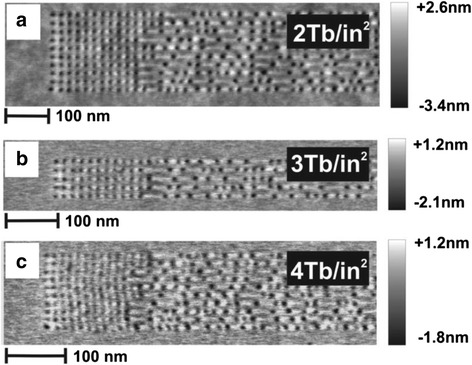
Topographic images of random data patterns used in the recording tests showing (**a**) 2 Tbit/in^2^ on a normal spin coated samples, and (**b**) 3 Tbit/in^2^, and (**c**) 4 Tbit/in^2^ on an ultraflat templated sample. Reprinted with permission from [17]

The ability to simultaneously record and read bits using tip arrays obviously endows thermo-mechanical probe memory with some advantageous traits such as ultra-high recording density and high data/write speed when in comparison with conventional data storage devices [13–16]. In addition, the resulting density can be further boomed by reducing the bit pitch that is determined by the rim size of the written bit [17]. It has been found that either using a tip with small contact radius or reducing the indentation depth can effectively compact the rim size, and therefore allow for higher recording density. These exciting findings and the inherent advantages of device itself have recently led to some remarkable technological advances on thermo-mechanical probe memory. In perspective of the storage medium, a Diels-Adler (DA) polymer that has a highly cross-linked, high-molecular weight at low temperature was proposed [18]. This polymer has a high wear resistance with good thermal stability below the transition temperature, whereas it turns out to be easily deformable and gentle on the tip above the transition temperature. The feasibility of using DA polymer to achieve 1 Tb/in^2^ has been demonstrated [18]. In addition to DA polymer, a polyaryletherketone (PAEK) polymer that unites phenyl-ethynyl groups in the backbone with diresorcinol units in the backbone for control of the glass-transition temperature was considered as another promising storage medium for thermo-mechanical probe memory due to its low glass-transition temperature less than 160 °C in the cross-linked state and superb thermal stability up to 450 °C [17]. These exceptional characteristics would give rise to a writing of indentation on a microsecond time scale as well as an effective minimization of thermal degradation for indentation with a hot tip. The capability of using a PAEK polymer templated on a cleaved mica surface to provide an areal density of 4 Tb/in^2^, the endurance of 108 write cycles, and data retention time of 10 years at 85 °C has been demonstrated [19–21], as illustrated in Fig. 5. The possibility of achieving 9 Tb/in^2^ based on the PAEK polymer was also theoretically predicted [22]. Another prospective material for thermo-mechanical recording as an alternative to polymer is NiTi shape-memory alloys (SMAs) [23]. NiTi is more immune to the normal environment effects at the shape-memory effect’s normal operation temperature than the poly (methyl methacrylate) (PMMA) polymer that is apt to charring at the temperatures around 350 °C. Besides, the low operating temperature and high thermal conductivity of SMAs allows the device using SMAs to have a less energy consumption for writing and more rapid erasing operation than that with PMMA polymer. The practicality of using SMAs as the storage medium for thermo-mechanical probe storage to achieve 0.5 Tbit/in^2^ has already been proven [23].

As thousands of cantilevers scan repeatedly back and forth across the medium surface, the tip and sample wear become more evident than other storage devices, thus exacerbating the lifetime of the thermo-mechanical probe device as well as the resulting density. In order to suppress the wear from media’s point of view, one possible approach is to introduce a photo-resistant layer between the silicon substrate and the PMMA polymer [12]. From the perspective of tip, tip wear can be aggressively mitigated by coating the tip with some hard materials. Recently, a moulded DLC tip designed based on atom by atom attrition mechanism was reported to give approximately four orders of magnitude improvement on wear compared to the carbon nanotube tip [24]. Such an improvement can be further enhanced by the use of SiC tip [25]. Another possibility to alleviate the tip wear is to actuate the tip with a periodic force at frequencies at or above the natural resonant frequency of the cantilever [26, 27]. In practice, it is preferable to operate AFM at intermittent-contact mode that can effectively reduce tip wear. However, the intermittent-contact mode that usually requires high cantilever stiffness is usually contradictory with the feeble cantilever used in thermo-mechanical storage to allow easy electrostatic actuation [28]. This can be solved by either utilizing amplitude modulation of the cantilever through electrostatic actuation [26] or slightly modulating the force on the tip-sample contact [28], allowing for an areal density of 1 Tb/in^2^ attained even after a sliding distance of 140 m.

### Magnetic Probe Memory

**Fig. 6:**
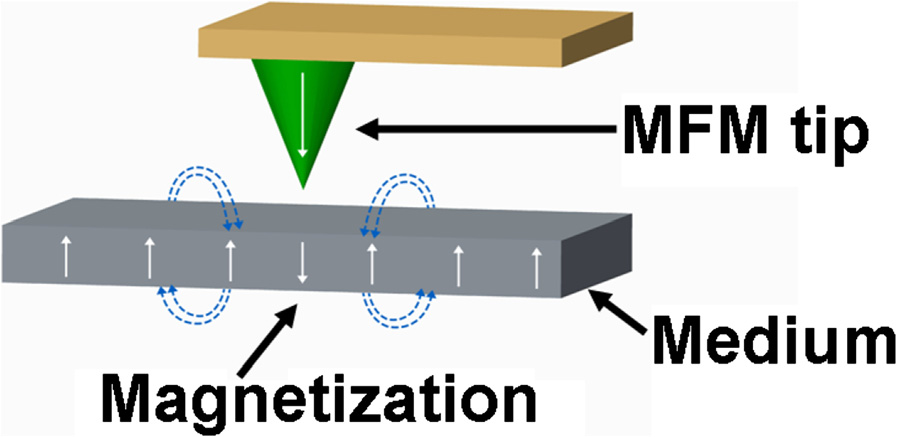
Magnetic probe memory system

**Fig. 7:**
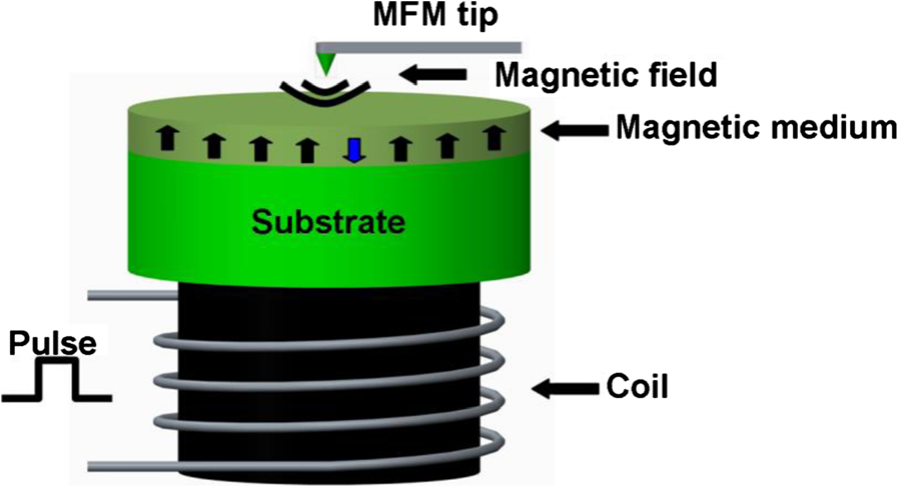
Magnetic probe memory using an external field

**Fig. 8:**
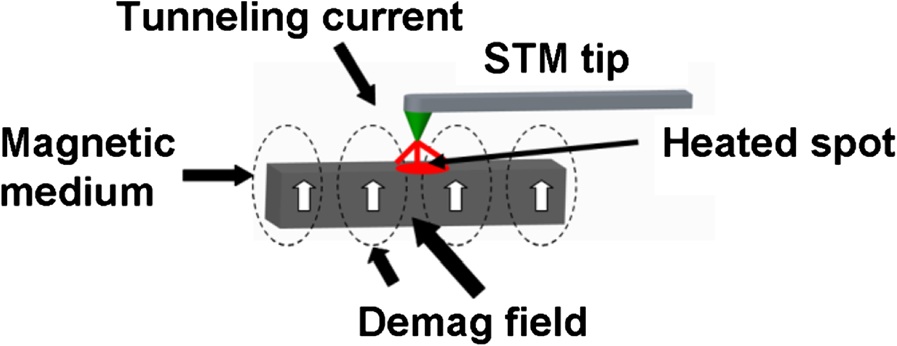
Magnetic probe memory using STM

Magnetic probe memory exhibits an analogous recording/readout mechanism to the conventional HDD. A probe tip from magnetic force microscope (MFM) scans over the surface of the magnetic medium whose magnetization can be reversed by the magnetic field emanated from the magnetic tip, to accomplish the recording operation, as illustrated in Fig. 6. During readout mode, the MFM tip is brought in close to the previously written magnetic dots and thus deflected up and down due to the magnetic force existing between the tip and magnetic dot. Such a variation of the position of MFM tip can be detected as the readout signal. It is well-known from the Neel-Arrhenius relation that the ratio of the product of the crystalline anisotropy and the volume of the bit to the product of the Boltzmann constant and the absolute temperature should be greater than 50 so as to maintain the thermal stability of the magnetic dots [29]. In this case, the reduction on the bit volume for higher density would require a larger crystalline anisotropy, thereby requiring a much higher writing field from magnetic tip. Nevertheless, due to the low field emanated from the magnetic tip, the crystalline anisotropy of the storage medium for magnetic probe memory needs to remain low to ensure the successful magnetization reversal while at the expense of the resulting recording density. For this reason, the magnetic probe memory has initially shown a relatively low recording density down to 200 Gb/in^2^, resulting from a vacuum MFM on Co/Pt multi-layered dots with perpendicular anisotropy [30, 31]. Fortunately, several methods to strongly enhance the writing field of MFM probes have been proposed recently, one of which is to applying an external field on a small coil mounted below the magnetic medium [32–38]. As can be seen from Fig. 7, the magnetization of the recorded film can be reversed by the superimposition of the writing field directly from MFM tip and the external field from the coil, leading to a density possibly up to 1.2 Tb/in^2^ by mean of a 30 nm tip radius [37, 38]. An alternative to achieve the magnetization reversal in the absence of the external field is to locally heat the medium using a tunneling current from scanning tunneling microscope (STM) probe to lower its crystalline anisotropy [39–43], as illustrated in Fig. 8. Thanks to this, the magnetization of the heated region can be switched by the demagnetization field of the surrounding film. This technique results in a variety of bit size on Co/Pt multi layers ranging from 800 to 170 nm probably due to the variations on medium itself and tip radius [44–48]. However, if the writing is performed using STM tip, the subsequent readout of the written bits can only be read by spin-polarized tunneling. In this case, MFM tip that is operated in field-emission mode is implemented instead of STM, by which a minimum bit size with 80 nm is secured with the assist of a pulse background field [32]. In addition to the methods above, such as heat-assisted magnetic probe recording technology can be realized by an incorporation of a heated AFM tip with a Co/Pt multilayer patterned medium prepared by sputtering on pillars of 90 nm diameter [49], whereas the readout manner for this technique remain unreported. Such a nano-indentation technique was recently applied to a ferromagnetic shape memory alloy (FSMA) to achieve four magnetic-based memory states using MFM probe due to magnetic field or stress-induced twin rearrangement along two crystal orientations [50].

In terms of readout process, a short tip-sample distance on the order of 10 nm or less is required in order to obtain a readout image with high resolution. However, for MFM tip operating within such a small distance, non-magnetic tip-sample interactions play a more important role and would introduce unwanted topographic interference. This drawback can be overcome by integrating a magneto-resistive sensor on the magnetic tip [51–55]. A cantilever integrated with a spin-valve sensor was recently reported, and leads to a resolution around 1 μm, which is somewhat inadequate for probe-based storage [56]. However, it is possible to incorporate the magnetic field sensor used for modern HDDs that can readily give a resolution below 20 nm with the magnetic probe memory. In addition to this, another novel approach making use of dual tips for both topography and magnetic field imaging has been recently proposed [57]. According to this method, a cantilever is cut in half using the focused ion beam technique, whereby two different tips, one magnetized tip and one non-magnetized tip, are implemented to tackle the topography imaging and magnetic imaging, respectively. This dual-tip technique can radically suppress the perturbations of the magnetic tip as compared to the standard MFM methods.

### Ferroelectric Probe Memory

**Fig. 9:**
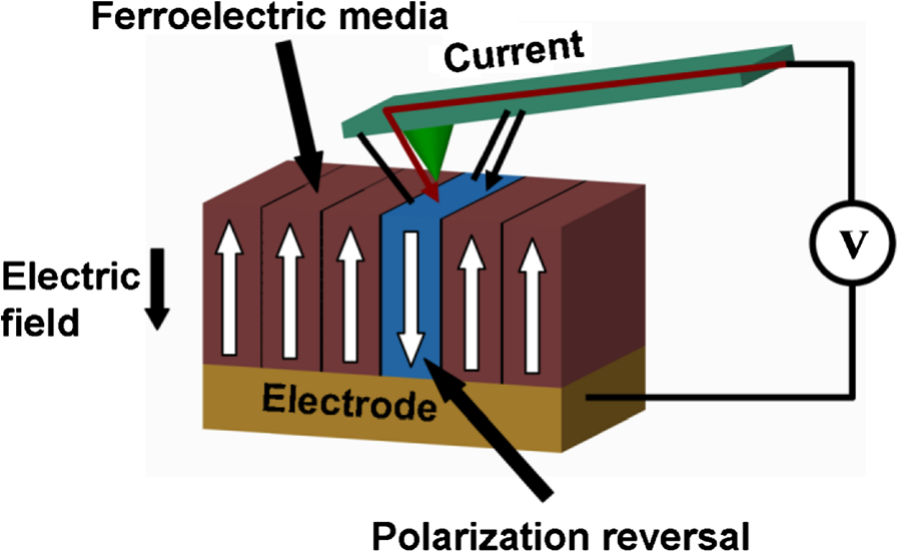
Ferroelectric probe memory system

**Fig. 10:**
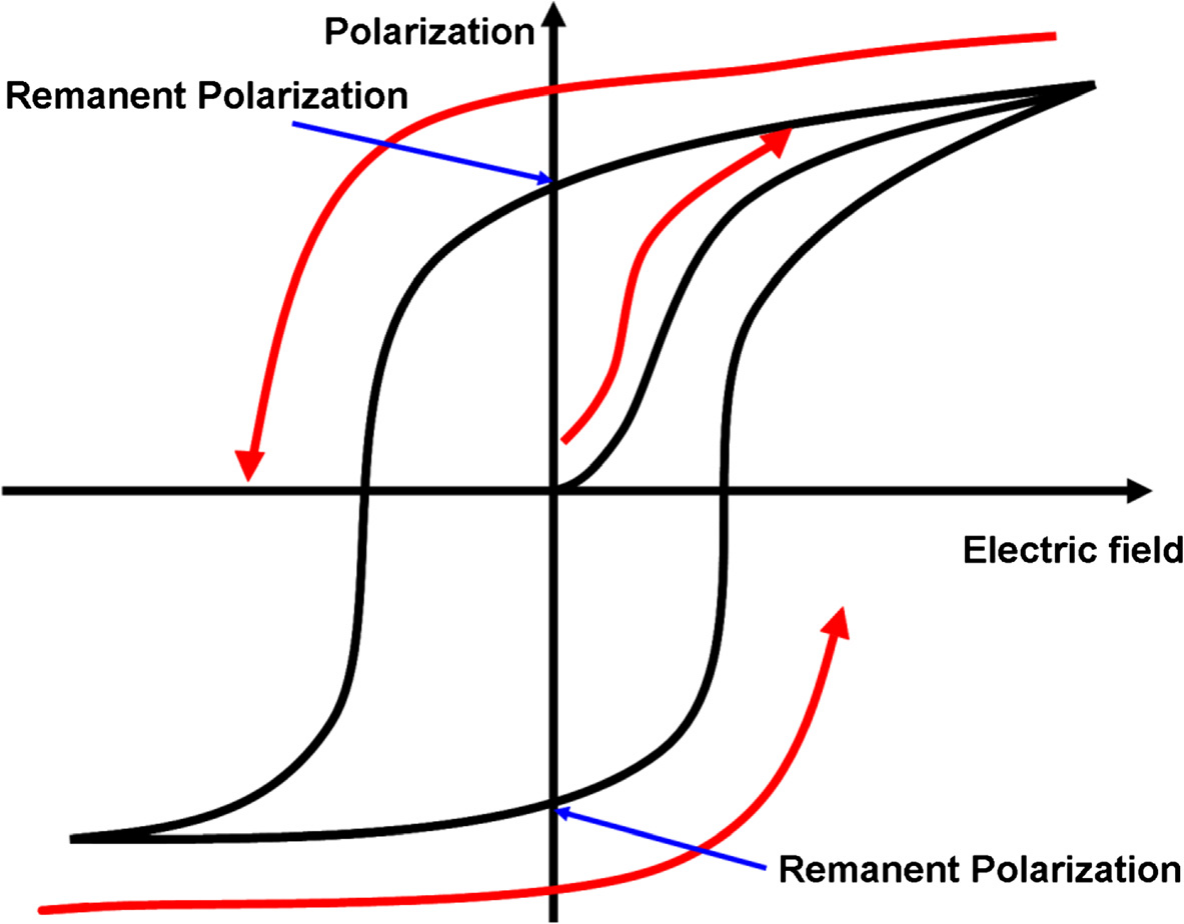
Hysteretic loop of FeRAM

The storage medium of ferroelectric probe memory is ferroelectric material showing a non-linear relationship between the applied electric field and the store charges. As illustrated in Fig. 9, applying an external electric field to the ferroelectric material can align its dipole with the field direction due to the small shifts in the position of atoms associated with shifts in the distributions of electronic charge in the crystal structure, whereas removing the electric field would drive dipole back to its original polarization state. Such a relationship can be simply interpreted by a hysteretic loop, as shown in Fig. 10. Therefore, these two distinct polarizations of dipole can be used to represent binary digits. During the recording process, a conductive probe where an external field is applied is brought in contact with the ferroelectric medium whose polarization can be switched according to the direction of the applied field. The readout process of ferroelectric probe memory adopts a very different mechanism from other probe storage memories, as its readout operation can only be conducted under “0” state. Consequently, no readout signal is detected for the case of reading “0” state. In contrast, to read “1” state, a current pulse is introduced to induce the polarization re-orientation, which is regarded as the readout signal. The readout process of ferroelectric probe memory apparently requires an erasing of the previously written data, which is known as “destructive readout” [58]. It should be kept in mind that ferroelectric materials, exemplified by lead zirconate titanate (PZT), has exhibited much higher energy density than the highest ever reported energy density for magnetic materials [59, 60]. Thanks to this, an unwanted thermal fluctuation effect induced by ambient thermal energy for ferroelectric materials would occur at a much higher density limit than magnetic materials. The density up to 3.6 Tb/in^2^ on an atomically smooth PZT medium has been achieved [61]. The contrast in the positive and negative poling region can be significantly mitigated by either carefully controlling the deposition temperature or using the PZT films exposed to an ion beam using an electron cyclotron resonance (ECR) ion source with an argon and oxygen gas mixture [62]. The minimum single-digit domain with good stability in PZT film was recently reported to 4 nm in diameter, suggesting for a recording density of 40 Tbit/in^2^ [63]. Another promising ferroelectric material in addition to PZT is LiTaO_3_ that shows unique electro-optical properties as well as good mechanical and chemical stability [64–69]. The demonstration of accomplishing 10 Tbit/in^2^ and 13 Tbit/in^2^ using thin LiTaO_3_ single-crystal films and a background field has been recently reported [70, 71]. For the latter case, data retention was measured by investigating the readback signals at elevated temperatures, and an activation energy of 0.8 eV at an attempt frequency of 200 kHz was found, sufficient for a data retention of 10 years [72].

Although the ferroelectric probe memory exhibits a capability of providing densities of multi-Tbit/in^2^, its destructive readout mechanism requires data to be refreshed after readout and this would cumulate read/write cycles and eventually causes severe fatigue. In order to overcome the adverse effects of the destructive readout, several non-destructive readout methods have been recently proposed including piezoelectric force microscopy (PFM) readout [73–75] and scanning nonlinear dielectric microscopy (SNDM) readout [64, 76–78]. For PFM readout, a conductive probe where a small AC tip-sample voltage is applied is brought in contact with the sample, and the response of the probe related to the polarization state of ferroelectric domains is detected by PFM as a readout signal at a frequency below the cantilever resonance. It should be pointed out that the resulting electric field would possibly change the permittivity of the ferroelectric domain, thus resulting in an advent of second harmonics [73]. An alternative method to operate probe in non-contact mode is SNDM readout that possesses from a fact that the reversal of the ferroelectric polarization can slightly change the storage medium’s capacitance due to the non-linearity in the permittivity tensor [79]. The variation on the capacitance would slightly change the resonance conditions that can be detected by monitoring the cantilever vibration if the cantilever is excited with a fixed AC voltage by means of a lock-in technique. Another method reported the direct piezoelectric effect to build up charge on the tip as a result of the tip-sample load force [78]. The resulting current is proportional to the load force, leading to a trade-off with endurance, as tip wear increases with the load force.

### Phase-Change Probe Memory

**Fig. 11:**
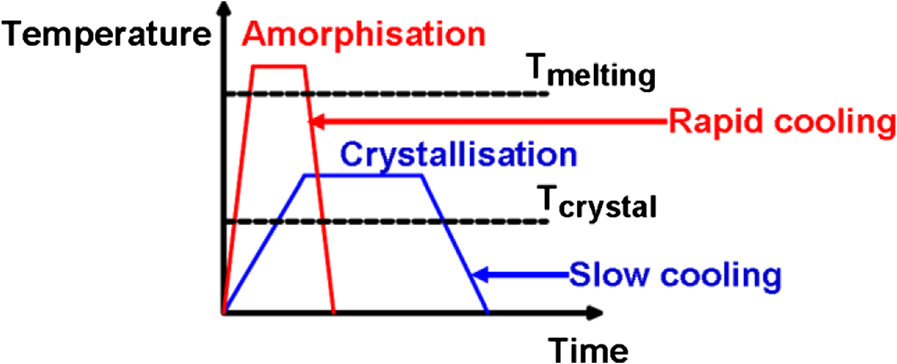
Thermal switching of phase-change materials between amorphous and crystalline phases. *T*_*m*_ is the melting temperature and *T*_*crystal*_ is the crystalline transition temperature

**Fig. 12:**
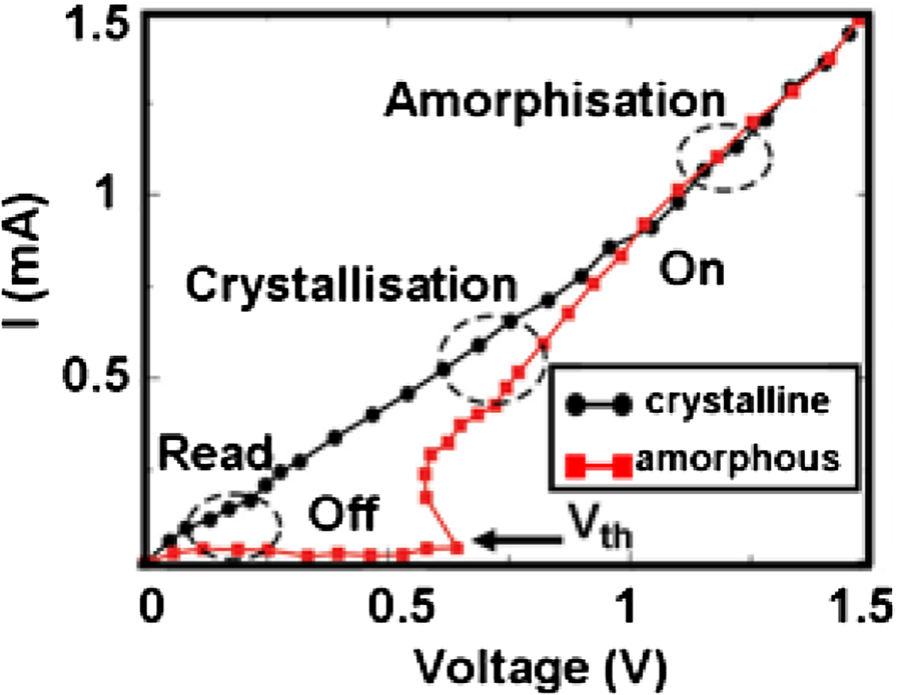
Threshold switching phenomenon observed in phase-change materials. *V*_*th*_ is the threshold-switching voltage, and *I* represents the resulting current. Reprinted with permission from [82]

Although the history of phase-change probe memory is fairly shorter than its compatriots, it has been considered as one of the most promising mass storage devices for the next generation due to its potential for ultra-high density, high data rate, long data retention, and low cost. Its advantageous characteristics arise from the combination of phase-change materials with nanoscale-conductive probe. Phase-change materials, mainly represented by chalcogenide alloy, are capable of exhibiting two distinct physical phases depending on the arrangement of the atomic structure [80], i.e., amorphous phase and crystalline phase. The atomic structure of amorphous phase presents a short range translational order, while for crystalline phase, the atoms are distributed in a repeating or periodic array over large atomic distances. Such a structural variation makes the electrical and optical properties of amorphous phase significantly different from the crystalline phase, as the phase-change materials in crystalline phase normally shows much lower electrical resistivity and higher optical reflectivity than those of amorphous phase. Another intriguing feature of phase-change materials is that its two distinct phases can be switched between each other [81]. In order to generate crystalline phase, phase-change materials under amorphous phase needs to be heated above glass transition temperature but below the melting point, followed by a slow cooling to drive materials back to the energetically more favorable crystalline phase. To recover amorphous phase, phase-change materials in crystalline phase is required to be heated above melting temperature, subsequently followed by a fast quench to prevent the re-crystallization. Such a reversible transition is illustrated in Fig. 11. In addition to the thermal switching, a unique electronic switching phenomenon, known as “threshold switching”, is also found on phase-change materials [82], which is depicted in Fig. 12. As can be seen from Fig. 12, a high-resistance state (OFF state) is initially presented in the phase-change materials along with the increase of the bias voltage, while a sudden “snap back” takes place when the bias voltage reaches a so-called threshold voltage. Under this circumstance, a sharp increase on the resulting current is monitored, leading to a low resistance state (ON state). Threshold switching is highly important for phase-change materials, as it allows for the phase transition at relatively low voltage. Otherwise, phase transition process can only be induced using very high voltage, thereby causing unnecessary energy consumption. Several theoretical models have been proposed to interpret the threshold switching from different scientific perspective. The threshold switching was first ascribed to the thermal breakdown effect in the amorphous film [83], whereas later, more evidence revealed that threshold switching was mainly dominated by electronic mechanism [84, 85]. According to this, two different electronic models based on trap-limited theory [86] and a balance between a strong Shockley Reed Hall (SRH) recombination through trap levels and a generation phenomenon [87] were, respectively, speculated to be the origin of the threshold switching. Differing from above models, a crystallization model owes the origin of threshold switching to the field induced nucleation of conductive cylindrical crystallites [88]. To date, the physical mechanism that can explicitly explain the threshold switching behavior still remains unclear.

**Fig. 13:**
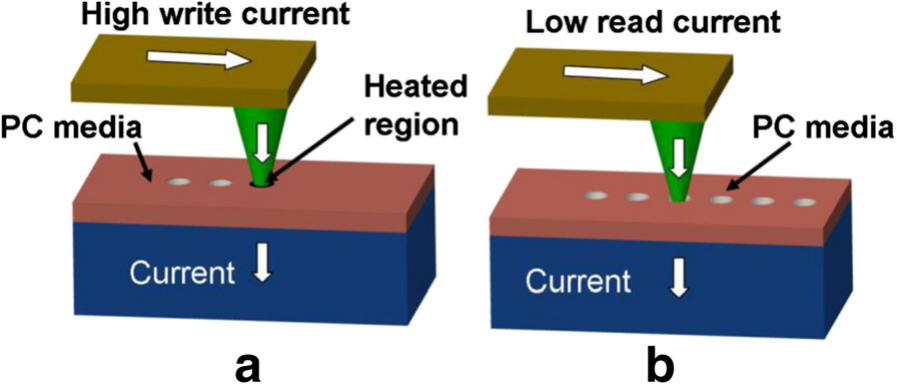
Phase-change probe memory when operated in **a** write mode, and **b** read mode

As phase-change materials in amorphous phase exhibit distinct electrical/optical properties from crystalline phase, it is viable for phase-change probe memory to utilize such properties difference to denote the binary digits “1” and “0”. In terms of phase-change probe memory, during the recording process, a write current is injected to phase-change media through the conductive probe tip to either heat the media to crystalline temperature for crystallization, or to the melting temperature followed by a rapid cooling to generate amorphization. The readout process is achieved by detecting the difference on the sensing current between highly conductive crystalline state and highly resistive amorphous state. The operations of phase-change probe memory are schematically shown in Fig. 13. As the conductive probe is employed as the conductive path for writing current, the dimension of the resulting phase-transition region would strongly depend on the radius of tip apex. In other words, it is viable to achieve multi-Tbit/in^2^ if the radius of the tip apex can be downscaled below around 20 nm [89]. In addition, the most commonly used storage medium in phase-change probe memory is Ge_2_Sb_2_Te_5_, usually referred to GST, which has been extensively adopted by rewritable optical disc and phase-change random access memory due to its capability of offering fast transition speed, long data retention time, and great endurance [90–93]. Given that the nanoscale probe tip and the GST medium are two key components of the phase-change probe memory, it is natural to infer that phase change probe-memory is capable of inheriting the performance superiorities of the nanoscale probe and the GST media.

**Fig. 14:**
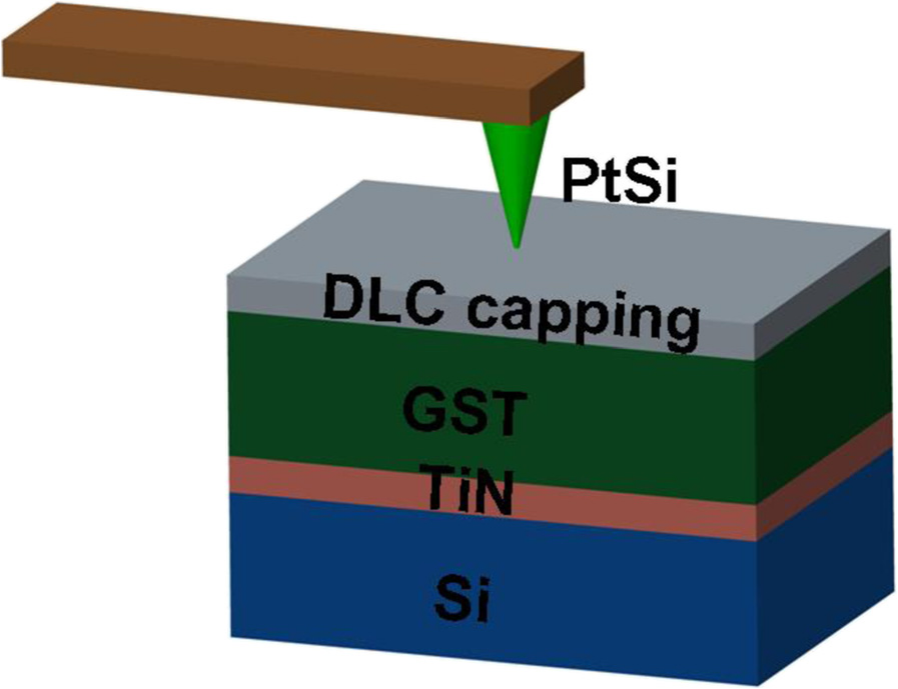
An optimized phase-change probe memory architecture

**Fig. 15:**
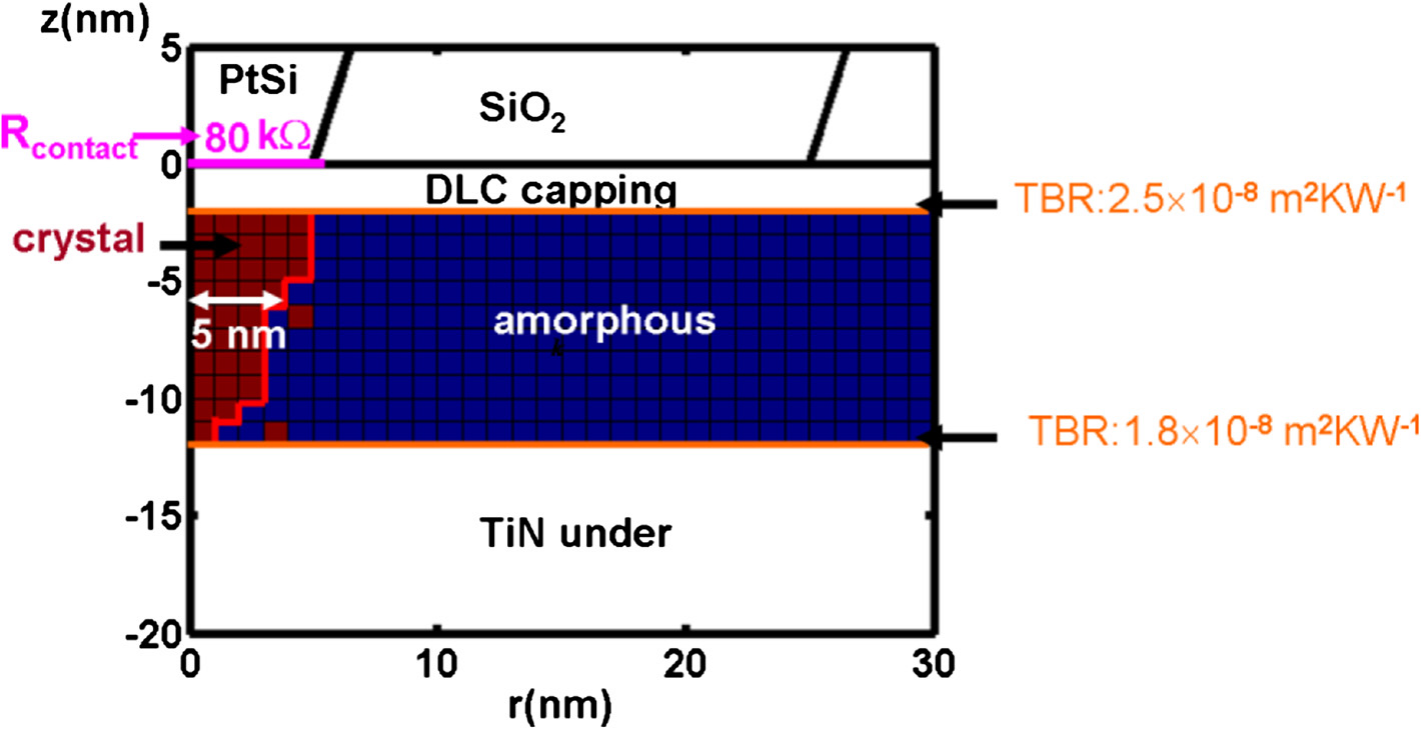
The resulting crystalline bit from the designed optimal electrical probe memory. The written bit exhibits a radius of approximately 5 nm, corresponding to 10 Tbit/in^2^

**Fig. 16:**
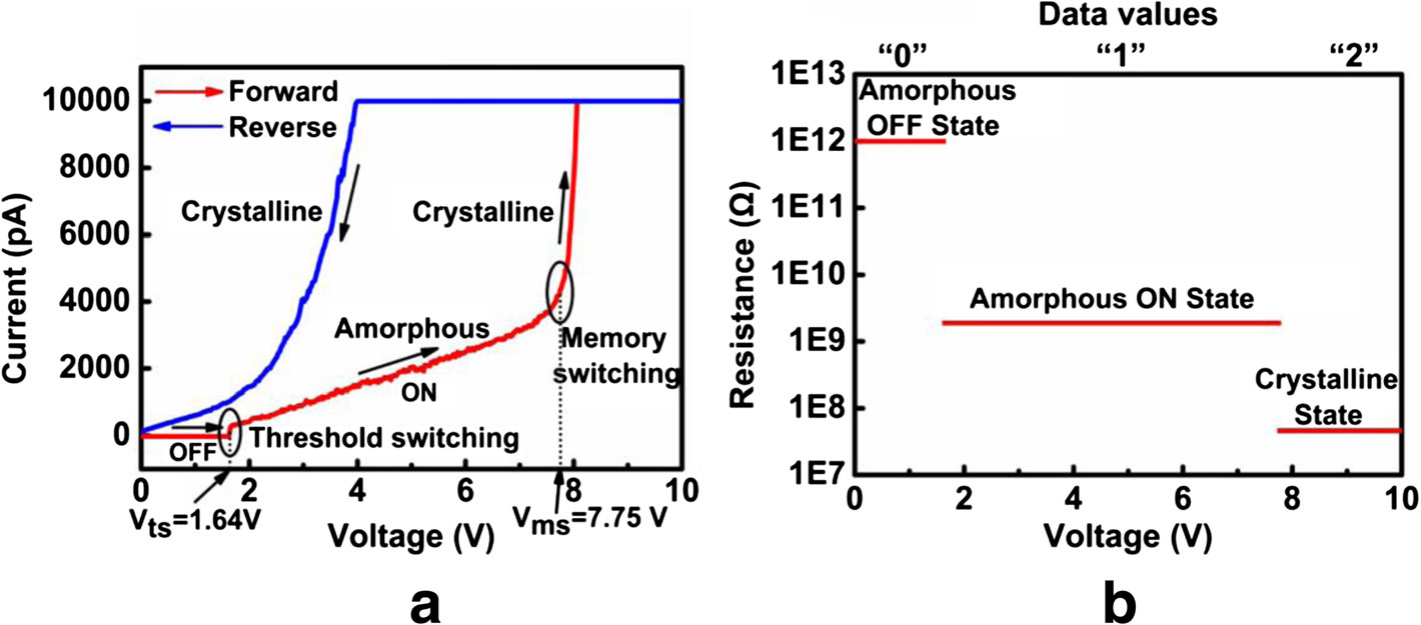
**a** Current-voltage spectrum of amorphous GST thin film measured in the range of 0–10 V with C-AFM; **b** the corresponding resistance of the amorphous OFF state, amorphous ON state, and crystalline state. Reprinted with permission from [111]

**Fig. 17:**
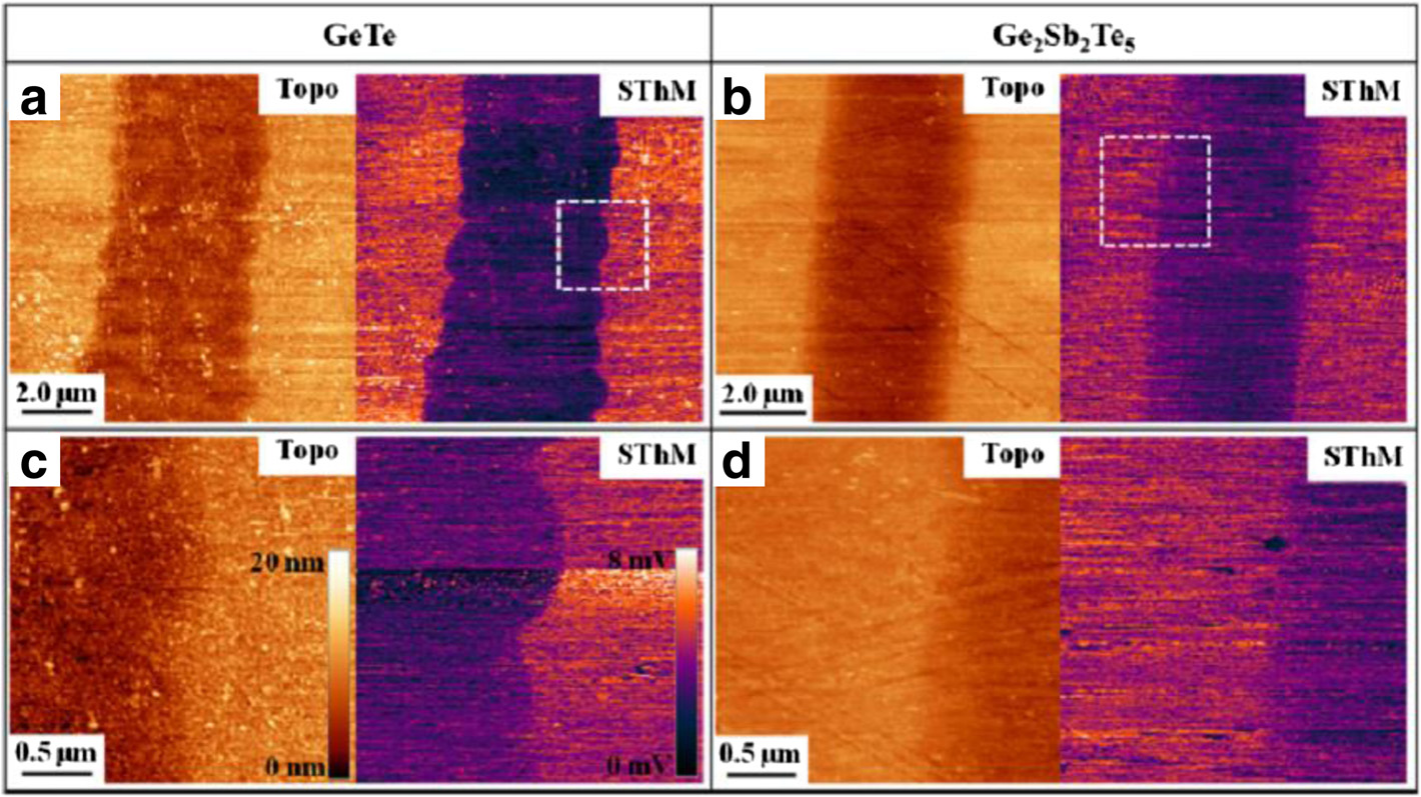
**a** Topography (*left sub-panel*) and SthM (*right sub-panel*) images with 10 μm and **b** 8 μm scan sizes, revealing a crystalline line written into 200 nm GT and GST amorphous thin films by a focused laser beam. **c** The 2.5-μm images for GT and (**d**) GST taken from the spatial locations marked by the insets in **a** and **b**. Reprinted with permission from [112]

Depending on the adopted architecture of phase-change probe memory, a variety of resulting bit size from 80 to 20 nm has been reported [94–100]. By the time of writing, the maximum areal density of phase-change probe memory that can be achieved experimentally is up to 1 Tbit/in^2^ [94] with corresponding power consumption of 100 PJ per bit [95], much lower than the thermo-mechanical probe memory. This is expected as phase-change probe memory requires the heating of bit volume only. Nevertheless, a new paradigm to induce the phase transition by a heated tip has recently been introduced [101], in which the AFM probe is heated to crystalline temperature either by a resistive heater or by a pulsed laser diode, and subsequently brought in contact with the GST to induce the phase transition. An areal density up to 3.3 Tb/in^2^ was obtained with the desired write speed of 50 Mb/s for one probe when using a spinning disk to position the medium [101]. In addition, it is viable to achieve high density recording using a novel recording scenario that the probe moves a small distance over the media and thus gives rise to a recorded mark with “arbitrary” length by making use of the inter-symbol-interference (ISI) effect, also known as mark-length recording. The advantage of mark-length recording over the conventional mark-position recording arises from the independence of the resulting density on the size of the tip apex, allowing for an increase of user densities by at least 50 % and potentially 100 % with an implementation of relatively blunt tip [102]. A typical phase-change probe memory usually consists of a nanoscale conductive probe and a phase-change media stack that comprises a GST storage layer sandwiched by a capping layer and a under layer, which are all deposited on silicon (Si) or silicon dioxide (SiO_2_) substrate. The role of capping layer is not only to protect GST layer from wear and oxidization, but also to build an effective electrically and thermally conductive path between probe tip and GST layer [103]. To satisfy the latter, the electrical conductivity of the capping layer needs to be relatively high in order to generate sufficient Joule heating inside the GST layer, while its thermal conductivity is required to be low to prevent heat from dissipating across the capping layer. However, a capping layer with high electrical conductivity is usually not desired as this would cause the presence of the short circuit during the readout process [104]. Compared with the capping layer, the under layer plays a less important role in determining the writing and readout performance of the phase-change probe memory, as it primarily acts as a bottom electrode. In this case, the electrical conductivity of the bottom layer is required to be much higher than capping layer so as to collect adequate write/readout current, while a relatively low thermal conductivity is preferable for the bottom layer to maintain sufficient Joule heat inside the storage layer [105, 106]. In addition, as both capping and bottom layers show thickness-dependent electrical/thermal conductivities [107, 108], their thicknesses also need to be carefully determined. Given the fact that threshold voltage is approximately equal to the product of layer thickness and threshold field, a thin GST is usually preferable so as to reduce the threshold voltage and thus the written power for a given low writing pulse. Therefore, an optimized design of the electrical probe memory device has been proposed by taking into account aforementioned factors as well as the underlying issues from practical fabrication process [109], as shown in Fig. 14. As can be seen from Fig. 14, this optimized phase-change media stack adopts a diamond-like-carbon (DLC) capping layer with thickness, electrical conductivity, and thermal conductivity of 2 nm, 100 Ω^−1^m^−1^, 0.2 Wm^−1^K^−1^, and a titanium nitride (TiN) bottom layer with thickness, electrical conductivity, and thermal conductivity of 40 nm, 0.5 × 10^7^ Ω^−1^m^−1^, 12 Wm^−1^K^−1^, respectively. The thickness of GST layer is fixed to be 10 nm, which is the typical thickness for phase-change probe memory [89, 96]. The write and readout performances of the designed probe memory was assessed by a previously developed electro-thermal model that is, however, supplemented with several advanced modeling techniques including threshold switching, electrical contact resistance at PtSi/DLC interface, and thermal boundary resistance (TBR) at the interfaces of DLC/GST and GST/TiN [109]. The crystallization process in this model is determined by simultaneously solving the Laplace equation, classical heat transfer equation, and the nucleation-growth equation [6, 58, 103]. Solving the Laplace equation results in an electric field distribution inside the GST active layer, which is subsequently adopted as the Joule heating source for the classical heat transfer equation. Then, the temperature distribution inside the GST layer can be calculated by solving the heat transfer equation, whereby the temperature dependent crystal fraction of the GST layer can be obtained according to the nucleation-growth equation. Note that writing amorphous bit using electrical probe usually requires much higher temperature inside the capping layer than crystallization, and this may strongly harm the thermal stability of the capping layer. Because of this, the experimental writing of amorphous bits using electrical probe seems more difficult than writing crystalline bit, and was reported to be realized only by the stacks without capping layer [95, 110]. Therefore, in this paper, we are only taking into account the writing of crystalline bits. Figure 15 shows a production of a spherical shaped bit with a diameter of around 10 nm by a 6 V pulse of 100 ns, corresponding to the areal density of 10 Tb/inch^2^ and the data rate of >Mb/s. Besides, the resultant writing power and writing energy are 0.016 mW and 1.6 pJ, respectively. This implies that the total writing energy required for using 100 and 1000 tips in parallel are 0.16 and 1.6 nJ, respectively, much lower than any other probe-based memories [11, 41, 67]. Most importantly, the crystalline bit shown in Fig. 15 is reported to be thermodynamically stable at the temperature below 550 °C, demonstrating its long retention time at archival temperature [89]. Meanwhile, the ability to achieve multi-level recording using phase-change probe memory was also demonstrated recently [111]. It was found that the resistance of the GST thin film can be toggled among three different states (high, intermediate, and low resistances) by carefully controlling the voltage between the conductive probe and the GST layer, as shown in Fig. 16. Therefore, three data values “0”, “1”, and “2” can be assigned to these three resistance states, respectively, allowing for multi-level operations in phase-change probe memory. In addition to the use of conductive AFM, scanning thermal microscopy (SThM) was recently reported to have the capability of measuring the thermal conductivities and thermal boundary resistances of GeTe (GT) and GST thin films [112, 113]. During the measurement, the SThM probe is repeatedly brought into and out of contact with material surface and the thermal properties of the probed materials can be determined by measuring the comparison of the heat flow from the SThM probe before and after the contact. According to such technique, it is possible to distinguish the amorphous phase from the crystalline phase by sensing the change in the thermal properties between amorphous and crystalline region, as revealed in Fig. 17. This finding also makes SThM as an alternative tool to provide bit write/read functions with spatial resolution down to the nanometer scale.

## Conclusions

Today, the maximum achievable density resulting from conventional storage forms is currently limited below 1 Tb/in^2^, and further density enhancement on these devices does not seem to be very optimistic provided that there are no innovative storage technologies emerging in the near future to break through their respective physical limits. Compared with the conventional storage devices, the practicality of using probe-based storage memories to achieve multi-Tbit/in^2^ has been demonstrated on different probe storage devices. The characteristic comparison for different probe-based storage memories is shown in Table 1.

**Table 1:** Performance comparison of various probe memories [121]

	Phase-change	Magnetic	Thermomech	Ferroelectric
Density	3*.*3 Tb/in^2^	60 Gb/in^2^	4*.*0 Tb/in^2^	4*.*0 Tb/in^2^
Est. Max. density	*≈*10 Tb/in^2^	*≈*100 Tb/in^2^	*≈*10 Tb/in^2^	*>*10 Tb/in^2^
Read speed per probe	50 Mb s^*−*1^	*<*10 b/s	40 kb s^*−*1^	2 Mb s^*−*1^
Write speed per probe	50 Mb s^*−*1^	*<*10 b/s	1 Mb s^*−*1^	50 kb s^*−*1^
Travel per probe	2*.*5 m	0*.*5 m	750 m	5000 m

**Fig. 18:**
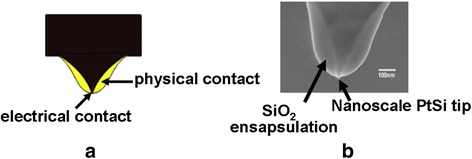
**a** Schematic of the “encapsulated” tip concept, and **b** the experimentally fabricated encapsulated tip. Reprinted with permission from [117]

The advent of thermal-mechanical probe memory has pioneered a novel application of probe on data storage field and has been considered as the most mature probe technology in comparison to its juniors. However, the energy consumption caused by thermo-mechanical probe memory seems to be problematic as the entire tip needs to be heated to perform write and readout process. This can be alleviated by a non-thermal writing mechanism that the AFM tip at room temperature is pressed into the polymer to generate the indentation [114]. This innovative writing strategy gives rise to a density up to 1 Tbit/in^2^ or more with an exemption from heating the tip [114]. Due to the superparamagnetic limit, the maximum areal density obtained from magnetic probe memory in the laboratory level is limited to a few of Gbit/in^2^, much lower than that of other probe storage memories. Moreover, the necessity to integrate a compliant cantilever with a sensitive force sensor for the readout of magnetic probe memory actually makes the array design more complicated. As a consequence, the practicality of magnetic probe memory is being strongly challenged, which may accelerate its demise in the future. Ferroelectric probe memory has recently received more attention than magnetic probe memory because of its higher density, faster readout speed, and more endurance cycles. In addition, several approaches aiming to reduce the tip wear have been recently examined. One possible way is to use a platinum iridium tip in the readout of ferroelectric media to increase the endurance of the tip [61]. It was found that no loss in either the read or the write resolution took place after a sliding distance of 5 km at 5 mm/s, resulting in an extremely low wear volume down to 5.6 × 10^3^ nm^3^. An alternative method to overwhelm tip wear is to implement a dielectric-sheathed carbon nanotube (CNT) probe with micrometer-long tips that can be immune to significant wear and ensure a high resolution for both write and readout processes [115]. Recently, a hard HfB_2_ coated tip was reported to extend the tip’s endurance beyond 8 km of sliding [116]. Benefiting from the merge of the nanoscale conductive tip and phase-change medium, the capability of phase-change probe memory to offer ultra-high density, fast write/readout speed, long retention time, and great endurance cycles has been demonstrated both experimentally and theoretically. A more attractive feature of phase-change probe memory than other probe storage devices arises from the fact that the conductive tip used in phase-change probe memory is only required to be electrically sharp rather than physically sharp. As a result, it is viable to fabricate an electrically sharp tip but with a large physical diameter, thereby mitigating tip tribology and wear issues without the sacrifice of the resulting density. Such an idea has indeed been experimentally demonstrated by the fabrication of a SiO_2_-encapsulated silicon tip with platinum silicide (PtSi) at the tip apex [117, 118], as shown in Fig. 18. Forming PtSi at the tip apex can significantly improve the conduction and wear properties of the conductive tip, due to its ability to provide superior anti-wear characteristics and very high electrical conductivity. In addition, the use of the dielectric encapsulation (SiO_2_) can increase the physical diameter of the tip and thus contribute to lower levels of wear due to the reduced pressure between tip and sample. Therefore, the integration of highly conductive PtSi tip apex with a non-conductive SiO_2_ encapsulation assigns such an encapsulated tip a capability to present a very small electrical contact area with the media even if the tip itself is not physically very sharp, and consequently enables a high density writing and reading simultaneously with an extremely good anti-wear characteristic. Nevertheless, phase-change probe memory is severely limited by the difficulty of amorphizing crystalline regions of the storage medium [119, 120].
